# Synthesis of different types of nano-hydroxyapatites for efficient photocatalytic degradation of textile dye (Congo red): a crystallographic characterization

**DOI:** 10.1039/d3ra08527a

**Published:** 2024-04-16

**Authors:** Md. Kawsar, Md. Sahadat Hossain, Sumaya Tabassum, Newaz Mohammed Bahadur, Samina Ahmed

**Affiliations:** a Glass Research Division, Institute of Glass & Ceramic Research and Testing, Bangladesh Council of Scientific and Industrial Research (BCSIR) Dhaka-1205 Bangladesh shanta_samina@yahoo.com; b Department of Applied Chemistry and Chemical Engineering, Noakhali Science and Technology University Noakhali Bangladesh; c BCSIR Dhaka Laboratories, Bangladesh Council of Scientific and Industrial Research (BCSIR) Dhaka-1205 Bangladesh

## Abstract

The textile industry, a vital economic force in developing nations, faces significant challenges including the release of undesired dye effluents, posing potential health and environmental risks which need to be minimized with the aid of sustainable materials. This study focuses on the photocatalytic potential of hydroxyapatite together with different dopants like titanium-di-oxide (TiO_2_) and zinc oxide (ZnO). Here, we synthesized hydroxyapatite (HAp) using different calcium sources (calcium hydroxide, calcium carbonate) and phosphorous sources (phosphoric acid, diammonium hydrogen phosphate) precursors through a wet chemical precipitation technique. Pure and doped HAp were characterized *via* different technologies, which consist of X-ray diffraction (XRD), Fourier Transform Infrared (FTIR) spectroscopy, scanning electron microscopy (SEM), as well as UV-vis spectroscopy. The effectiveness of the synthesized photocatalyst was evaluated by its interactivity with synthetic azo dyes (Congo red). The photodegradation of Ca(OH)_2__HAp, CaCO_3__HAp, ZnO-doped HAp as well as TiO_2_-doped HAp, were obtained as 89%, 91%, 86%, and 91%, respectively. Furthermore, at neutral pH, TiO_2_-doped HAp shows the highest degradation (86%), whereas ZnO-doped HAp possesses the lowest degradation (73%). Additionally, various XRD models (Monshi–Scherrer's, Williamson–Hall, and Halder–Wagner methods) were employed to study crystallite dimension.

## Abstract 2

Exploration of the crystallographic parameters of various nano-hydroxyapatites for enhanced photocatalytic degradation of Congo red dye.

## Graphical abstract

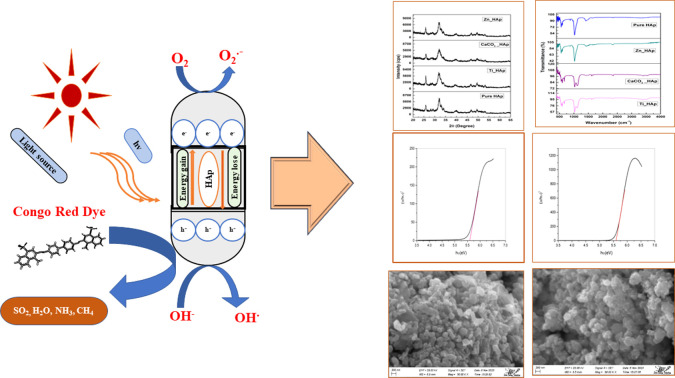

## 1 Introduction

Water pollution and the energy crisis are being addressed as global headaches and becoming more crucial events *via* population growth and fast industrialization. The worldwide production of organic dyes is approximately 450 000 tons, whereas 20–60% of wastewater is generated using hazardous organic azo dyes by numerous industries like pigment, fertilizer, cosmetics, paper, and textile.^1,2^ Synthetic dyes like Congo red (CR) are tough to biodegrade because of their stable molecules as well as complicated aromatic structures, which might induce carcinogenic and mutagenic effects.^3,4^ Several purification processes, such as reverse osmosis filtration, biological degradation, adsorption, flocculation, and centrifugation, were developed for wastewater purification.^5,6^ However, the majority of these purification techniques are plucked entirely for the degradation of the organic dye, owing to their high expenditure in operation, complex steps, and difficulty in applications.^7^ So, there is an urgent necessity to develop the existing technique that makes the purification process more efficient, easier, economical, and eco-friendly for synthetic dye degradation. Considering the above-mentioned criteria, the photocatalytic phenomena became a prominent innovation for the deterioration of synthetic dye along with various inorganic compounds like phenol and their derivatives.^8,9^ However, the degradation efficiency of dyes depends on the nature of the photocatalyst used. Because of this, several photocatalysts have been developed to estimate their performance in synthetic dye degradation.^10,11^ In the present, researchers have studied the photocatalytic capabilities of hydroxyapatite (HAp) by doping it with metals and evaluating its potential for photodegradation of dyes.^12–14^ Ideal HAp [Ca_10_(PO_4_)_6_(OH)_2_] crystal is monoclinic in nature. However, conventional HAp has a lattice deficiency, which consists of a hexagonal ionic compound along with a *P*6_3_/*m* space group, as well as 44 atoms in every repeating unit. The cations “Ca” are separate two distinctive sites such as Ca(i) and Ca(ii). Ca(i) is associated with the columnar site along with the *c* axis, where Ca(ii) is located around OH ions.^15^ Since HAp has a complex molecular arrangement, various types of cations can be incorporated into its crystal structure. Till now various types of cations were examined for replacing the “Ca” ions in crystalline HAp structure such as Ni, Ag/Fe, Co, TiO_2_, Ag, ZnO, Cds.^16–19^

Titanium dioxide (TiO_2_) and zinc oxide (ZnO), along with other semiconductors, have lately been used as affordable and environmentally safe photocatalysts for the decomposition of different contaminants.^20^ Conversely, ZnO has a significant advantage over TiO_2_ in terms of its ability to absorb a larger portion of the UV spectrum.^21^ HAp could lead to the generation of tailored materials with improved photocatalytic performance, crucial for environmental remediation and solar energy conversion.^22–24^ Though HAps are well known bioceramics, these can be utilized as the photocatalytic materials. HAp in pure form cannot impart so much catalytic activity but in doped form or with the combination other materials the photocatalytic activity is increased. Normally metals are used to replace the calcium ions of hexagonal crystal of HAp.

In this present work, HAp was synthesized from CaCO_3_ and Ca(OH)_2_ by wet chemical precipitation method. TiO_2_ and ZnO were used to replace the calcium ions to modify the crystallographic parameter with a hope to augment the photocatalytic activity. In-depth crystallographic analysis was also performed to find out the variation due to doping the materials.

## 2 Materials and methods

### 2.1 Materials

To proceed with this experiment, calcium carbonate (CaCO_3_) and calcium hydroxide (Ca(OH)_2_), as well as phosphoric acid (H_3_PO_4_), are utilized as potential sources for calcium and phosphate correspondingly. Apart from that, zinc oxide (ZnO), along with anhydrous titanium dioxide (TiO_2_), is employed as a dopant for synthesizing doped Hydroxyapatite. All these chemicals were purchased from E-Merck, Germany. No buffer solution was utilized in this experiment, but ammonium hydroxide (NH_4_OH) and nitric acid (HNO_3_) were used to maintain the pH (10–11) of the reaction media. The production of deionized (DI) water was simplified *via* a two-stage distillation procedure.

### 2.2 Methods

#### 2.2.1 Synthesis of doped and pure HAp

**Fig. 1:**
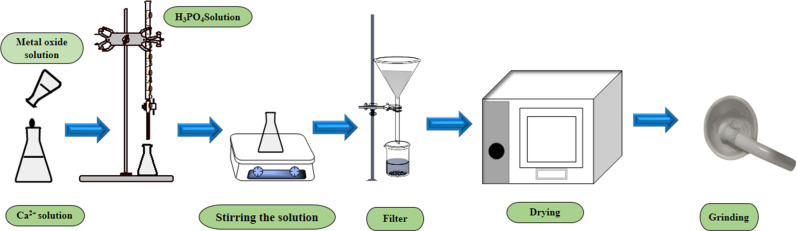
Systematic approach for pure and metal-oxide doped HAp synthesis.

To synthesize pure and metal-oxide doped HAp, the molar ratio of calcium and phosphate was maintained as 5 : 3. At the beginning, a predetermined amount of Ca^2+^, as well as TiO_2_ (0.05%) and ZnO (0.05%), are separately dissolved in 50 mL DI. Diammonium hydrogen phosphate [(NH_4_)_2_HPO_4_] and H_3_PO_4_ were then added into the different Ca^2+^ solutions [CaCO_3_ and Ca(OH)_2_] at the rate of 4 mL min^−1^. The solution is then stirred at 300 rpm while introducing H_3_PO_4_ and [(NH_4_)_2_HPO_4_] into the calcium-containing solution. Furthermore, the pH of the system was controlled at 10–11 by employing a 30% NH_4_OH solution. Finally, the solution was filtered and dried at 105 °C. A similar approach was conducted for metal-oxide-doped HAp (Fig. 1).

#### 2.2.2 Photocatalytic activity

**Fig. 2:**
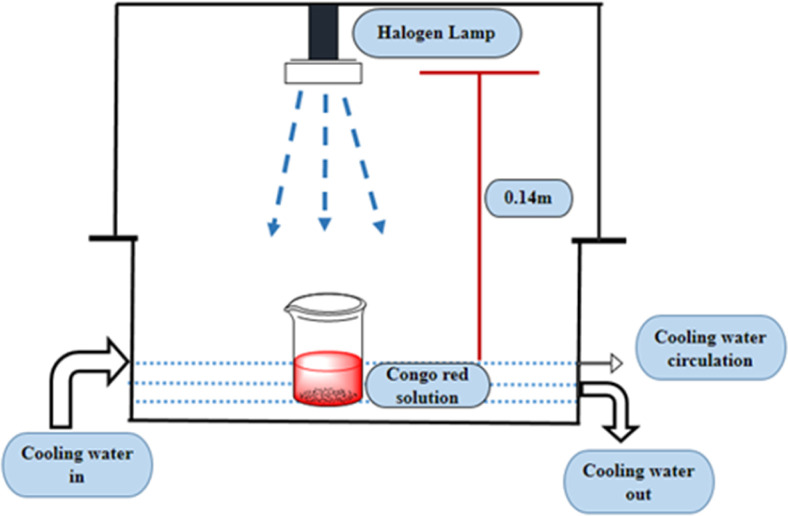
Laboratory setup for investigating photodegradation with halogen lamp.

The photocatalytic activity of pure and doped HAp was observed for the deterioration of Congo red dye (CR) solution under a halogen lamp (SEN TAI JM-500), which is placed on the top of the in-house built wooden box shown in Fig. 2. The distance from the lamp to the CR solution was kept constant (0.14 m). The box is then introduced into the cooling water circulation system. Additionally, the temperature and humidity of this system were maintained at approximately ∼25 °C and 60%, respectively. UV-vis spectrophotometer (Hitachi U-2910) was utilized to measure the absorbance of the dye solution by utilizing concentration. The degradation percentage (*D*_p_) as well as degradation capacity (*q*_e_) were measured by employing mathematical eqn (a) and (b).1
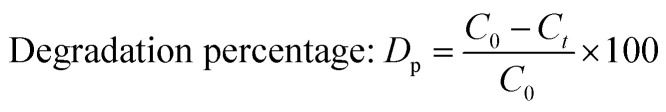
b
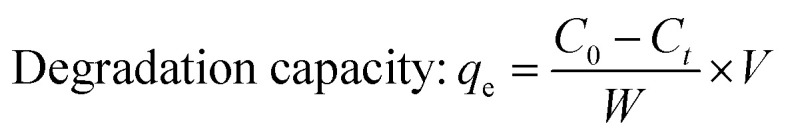
Here, *C*_*t*_ and *C*_0_ denote the final as well as initial concentration of the samples at time “*t*” correspondingly.^25^ In comparison, *V* and *W* represent the volume of the dye solution and the weight of the catalyst correspondingly.

#### 2.2.3 Scavenger's experiment

The captured test for radicals, and electrons was performed to examine the finest suitable species connecting photodegradation of dye under simulated sunlight (*i.e.* halogen lamp). Several scavengers were used in principal component analysis to measure the function of hydroxyl radicals, holes, and electrons.^26^ Using the above-mentioned ideal setting, isopropyl alcohol (IPA) as well as ethylenediaminetetraacetic acid (EDTA) were utilized to examine the function of hydroxyl radicals and electrons on all synthesized Hap. If not mentioned otherwise, 10 mL of the scavenger was chosen in 40 mL of 20 ppm Congo red dye for 90 min using 0.1 g of catalyst.

### 2.3 Crystallographic characterization *via* XRD

The crystallographic form of synthesized HAps was confirmed by employing an X-ray diffractometer (Model: Rigaku SmartLab SE), with scanning range at 2*θ* = 20–70°, as well as scanning step 0.01. The origin of radiation CuKα (*λ* = 1.54060 Å) was running within 40 kV and 50 mA, and cooling temperature was fixed at 19–20 °C. Finally, the observed phases were precisely estimated by assimilating them with a standard ICDD data sheet.

## 3 Results and discussion

### 3.1 XRD data interpretation

**Fig. 3:**
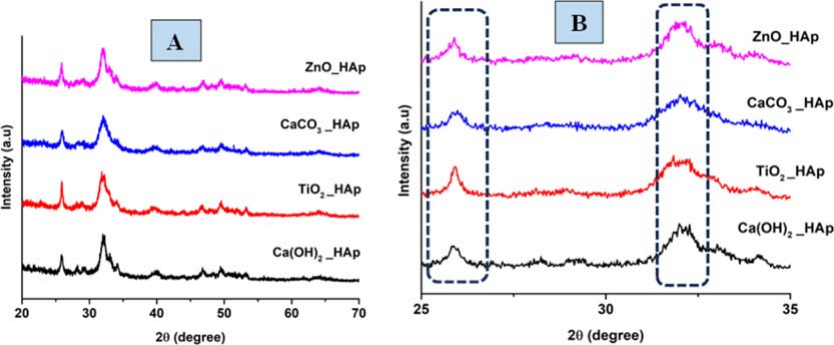
XRD patterns of pure and doped HAp (A) full scan, (B) focused region.

The patterns, obtained from XRD for doped HAp as well as pure HAp are exhibited in Fig. 3. The plane positions for these samples were visible at (002) 25.931°, (211) 31.831°, (112) 32.241°, (300) 32.961°, (202) 34.121°, (130) 39.881°, (222) 46.751°, (213) 49.531°. The HAps configuration has been verified by aligning *via* the published ICDD database (card no: # 01-074-0565), suggesting a hexagonal arrangement of crystals.

The crystallographic study assesses crystalline features such as cell volume, degree of crystallinity, dislocation density, lattice parameters, crystallinity index, microstrain, and crystallite size, employing eqn (1)–(7).^27,28^1
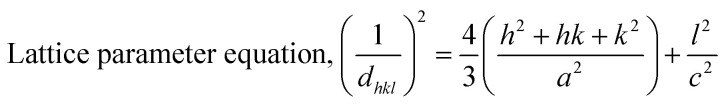
2
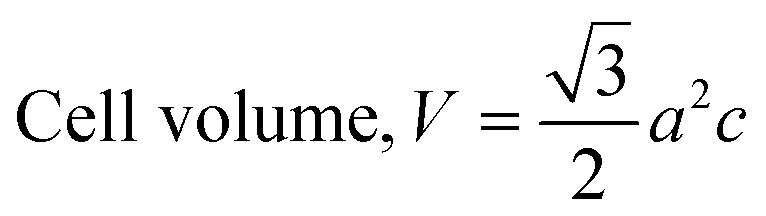
3
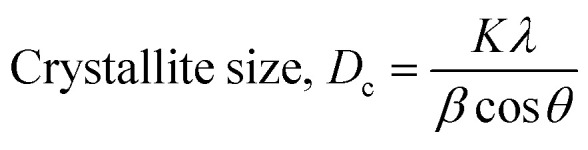

4 Microstrain, *ε* = *β*/4 tan *θ*

5
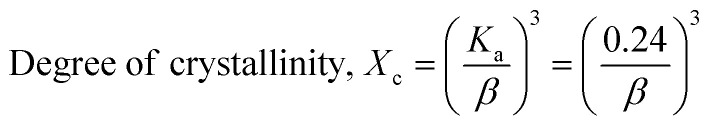
6
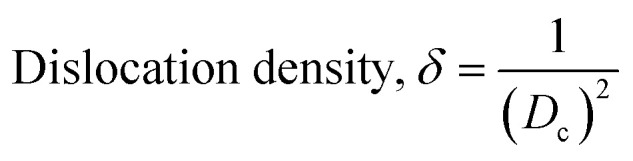
7
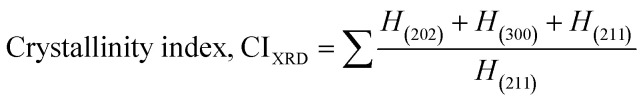


In the previously mentioned formulas, the unit cell is indicated by plane (*h*,*k*,*l*) and *a*,*b*,*c* reflects as lattice variable, *X*_c_ = crystallinity degree, *θ* = angles of the diffraction (in degree), *β* = FWHM (full width at half maximum) in radian, *D*_c_ = dimension of repeating unit, *δ* = dislocation density, *K* = shape factor (arbitrary constant)/Scherrer's constant = 0.94, *H*_(*hkl*)_ = peak height of the respective plane, *K*_a_ = 0.24, for HAp, as well as CI_XRD_ = crystallinity index. The specific surface area of the synthesized HAp was estimated through eqn (8). Where density as well as crystallite dimensions of HAp were denoted by *ρ* (3.16 g cm^−3^) as well as *D*_c_.^29^8
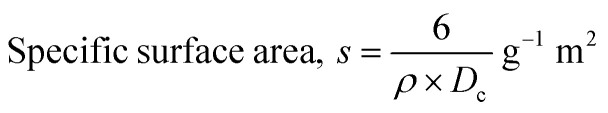


Crystallite dimensions in orderly distributed substances are essential in different uses, alongside minuscule crystallites being distinguished by enormous surface areas and *vice versa*.^30^

Microstrain leads to crystallite deformation, which results in changes in element's features, especially applicability. Imperfection in crystalline substances is brought about by defects such as point dislocation, line dislocation, and area dislocation, which have a strong connection to the structure of the crystal.^31^ The value of line dislocation was determined *via*eqn (6), and the date is shown in Table 1.

**Table 1:** Synthesized HAp and doped HAp crystallographic parameters

Parameter	Ca(OH)_2__HAp	TiO_2__HAp	CaCO_3__HAp	ZnO_HAp
Lattice parameter	*a* = *b* = 9.39, *c* = 6.86	*a* = *b* = 9.39, *c* = 6.87	*a* = *b* = 9.17, *c* = 6.86	*a* = *b* = 9.38, *c* = 6.87
Crystallite size, nm	10.20	7.17	6.56	9.07
Degree of crystallinity	26.012 × 10^−3^	9.04 × 10^−3^	6.91 × 10^−3^	18.344 × 10^−3^
Microstrain, *ε*	12.31 × 10^−3^	17.39 × 10^−3^	19.06 × 10^−3^	13.47 × 10^−3^
Dislocation density (×10^15^ lines per m^2^)	9.59	19.40	23.22	12.13
Crystallinity index, CIXRD	1.21	1.38	0.97	0.83
Specific surface area, *S* (g^−1^ m^2^)	0.186	0.26	0.28	0.020
Volume of unit cell (Å^3^)	524.33	525.26	499.69	523.86

The level of crystallinity significantly impacts the properties of materials, yet efficiently modulating it can be challenging. The investigation indicates that HAp exhibits levels that vary in the degree of crystallinity, while microstrain alludes to the intrinsic stress of crystalline planes, which can manifest as tensile or compressive forces.

The crystallinity index (CI) is explained for measuring the numerical quantification of crystal structure. In this segment, solely the (XRD) X-ray diffraction data were analyzed for calculating the crystallinity index by utilizing eqn (7), and the resulting values are shown in Table 1.

#### 3.1.1 Estimation of crystallite size using various models

Exact crystallite dimension estimation for any purpose is a vital requirement. Yet, for determining the size of the crystallite of HAp specimens, many approaches and algorithms have been developed, including the Williamson–Hall Method (WHM), Monshi–Scherrer Method (MSM), and Halder–Wagner Method (HWM). The Williamson–Hall Method was also expanded, focusing on the UDEDM (uniform deformation energy density model), UDM (uniform deformation model), and UDSM (uniform stress deformation model).

#### 3.1.2 Monshi–Scherrer method (MSM)

To measure the precise size of the crystal, Monshi–Scherrer's method, which is also known as the modified form of Scherrer's equation, is utilized. This revised equation was developed by taking “ln” on the side of Scherrer's equation, which is denoted in eqn (3). The mathematical formula of this modified form is expressed in eqn (9).^32^9
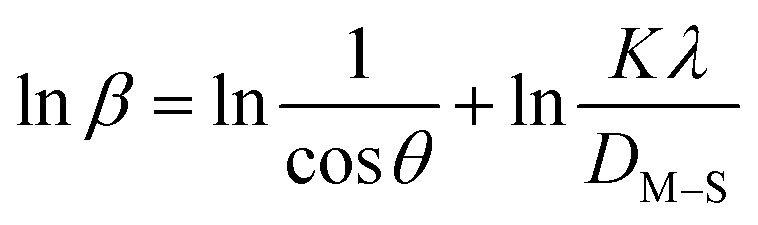


**Fig. 4:**
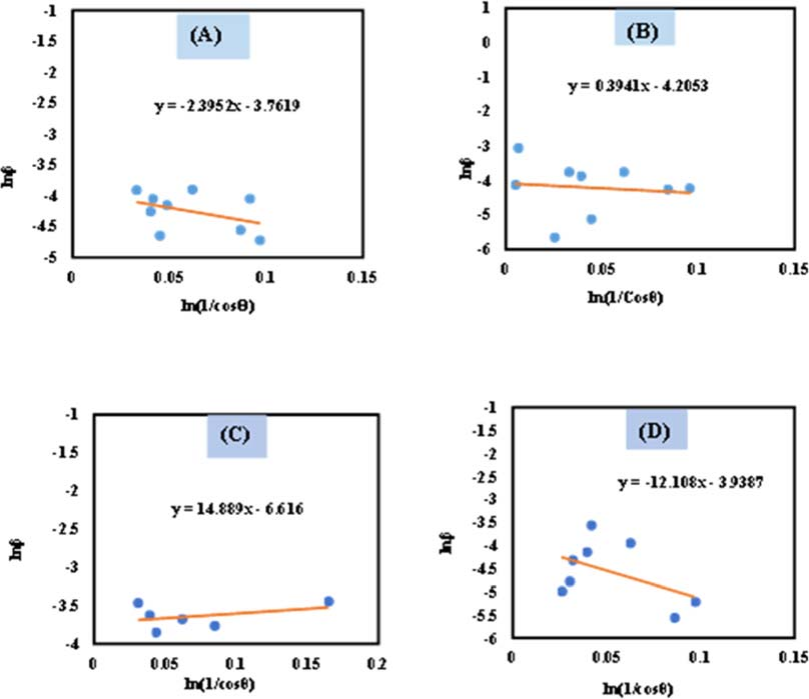
Determination of crystallite size by utilizing Monshi–Scherrer method equation for (A) Ca(OH)_2__HAp, (B) TiO_2__HAp, (C) CaCO_3__HAp and(D) ZnO_HAp.

For the implementation of the graph of this revised model, 
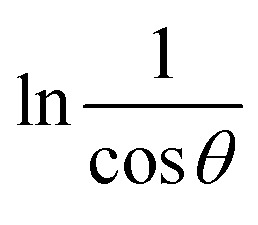
 was placed on the *X*-axis, and ln *β* was evaluated on the *Y*-axis (shown in Fig. 4). The straight-line equation (*y* = *mx* + *c*) and the eqn (9) were analyzed to estimate the slope, where the intercept was noted as 
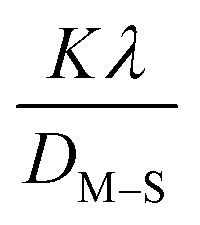
. This method supplied an indicator for the assessment of the reliability of results. Subsequently, the resultant magnitude of crystal size was obtained 5.96 nm for Ca(OH)_2__HAp, 9.29 nm for TiO_2__HAp, 103.56 nm for CaCO_3__HAp, and 7.12 nm for ZnO_HAp.

#### 3.1.3 Williamson–Hall method (WHM)

Scherrer's Equation, whereas resolving the impact of the Size of crystallite on XRD reflection, neglects the inherent strain in nanocrystals resulting from factors like dislocations, point defects, stacking, as well as boundaries of grains.^33^ The Williamson–Hall examination may identify an inherent strain, which can be determined by examining the effect of strain on crystallite dimension data. Eventually, the overall broadening may be described as eqn (10).^34^

10 *β*_Total_ = *β*_size_ + *β*_strain_

where *β*_strain_ is connected to the strain broadening effect as well as *β*_size_ is the broadening due to its size. The modified form of the Williamson–Hall, considered a UDM, USDM, and UDEDM, will be discussed in this context.^35^

#### 3.1.4 Uniform deformation model (UDM)

The estimated value of strain derived through crystalline defects and deformation in the synthetic HAp can be mathematically represented as a eqn (11).^36^11
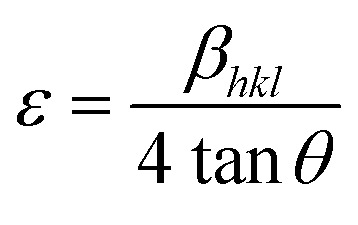


The UDM idea relies on the concept of homogenous strain in all directions and regards lattice strain as isotropic in nature despite its spatial amplitude.^37^ The peak broadening, which is caused *via* lattice strain, is often denoted in eqn (12).

12 *β*_strain_ = 4*ε* sin *θ*

The overall broadening, *β*_*hkl*_ is expressed as FWHM of a reflected peak, which is related to the influence of the strain of crystal lattice (*β*_strain_) and the value of the size of the crystals (*β*_size_) in a specific peak that may be stated as eqn (13)–(15).

13 *β*_*hkl*_ = *β*_size_ + *β*_strain_

14
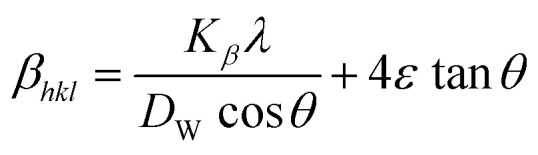


Eqn (14) can be written as:15
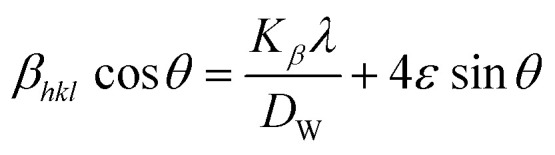


**Fig. 5:**
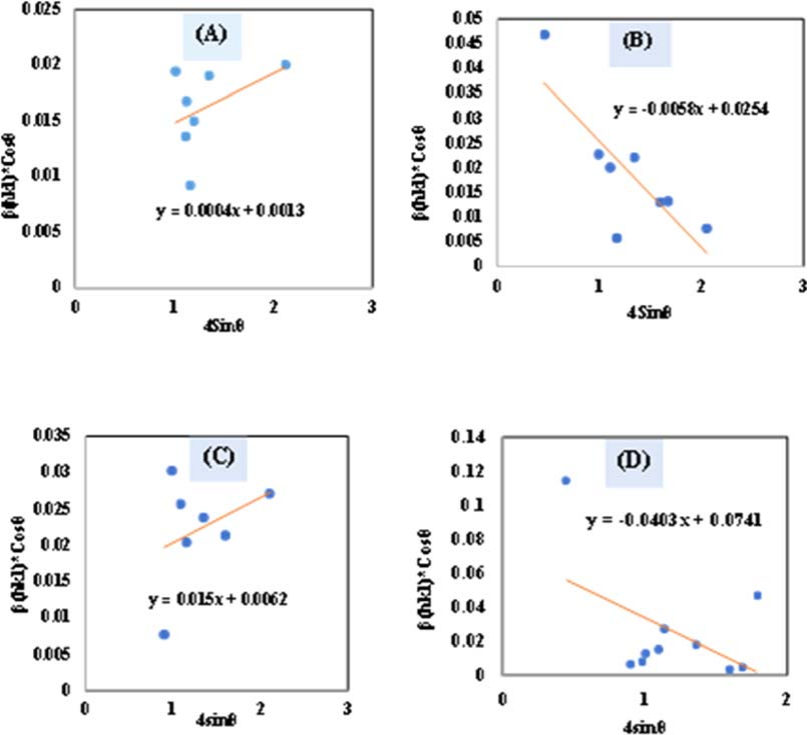
Determination of crystallite size by utilizing uniform deformation model for (A) Ca(OH)_2__HAp, (B) TiO_2__HAp, (C) CaCO_3__HAp and (D) ZnO_HAp.

Placing 4 × sin *θ* along the *X*-axis as well as *β*_*hkl*_ × cos *θ* on the *Y*-axis permits a straight-line equation. In the graph, both slope (*ε*) and crystallite size (*D*_w_) (*y*-intercept) can be computed. The graphs are depicted in Fig. 5, and the computed (*D*_w_) and *ε* values are reported in Table 2.

**Table 2:** Microstructural characteristics of hydroxyapatite utilizing various models in this study

Model name		Crystallite size (in nm), strain, *ε*, stress, *σ* (in N m^−2^), energy density, *u* (in J m^−3^)			
		Ca(OH)_2__HAp	TiO_2__HAp	CaCO_3__HAp	ZnO_HAp
Monshi–Scherrer's method		5.9662	9.295	103.56	7.120
Williamson–Hall method	UDM	*ε* = 0.0004, *D*_w_ = 106.656	*ε* = 0.0058, *D*_w_ = 5.4588	*ε* = 0.015, *D*_w_ = 22.36	*ε* = −0.0403, *D*_w_ = 1.87
	USDM	*σ* = −147237, *D*_(*hkl*)_ = 2.4848	*σ* = −35098, *D*_(*hkl*)_ = 5.45	*σ* = 90 117, *D*_(*hkl*)_ = 22.36	*σ* = −171655, *D*_(*hkl*)_ = 2.236
	UDEDM	*u* = 1.80 × 103, *D*_w_ = 2.48	*u* = 835.730, *D*_w_ = 3.6392	*u* = 8.1 × 10–11, *D*_w_ = 22.3635	*u* = 539.12, *D*_w_ = −1386.54
Halder–Wagner method		1.468	3.1746	1.11	1.2674

#### 3.1.5 Uniform stress deformation model (USDM)

UDM models, depending on material homogeneity and isotropic nature, frequently remain unvalidated because of the potential anisotropic nature of real crystals, requiring an altered Uniform Stress Deformation Model (USDM).^33^ From Hooke's law, it is known that there is a linear link between *ε* (Strain) as well as stress (*σ*) expressed *via*eqn (16).

16 *σ* = *Y*_*hkl*_*ε*

The mathematical expression *Y*_*hkl*_ depicts Young's modulus or modulus of elasticity, which is a reliable approximation for minimal strain. Increasing the amount of strain causes a shift in the magnitude of Young's modulus, demonstrating that the strain is not linear in nature.^38^ By rearranging and replacing eqn (16) with eqn (10), we have the following relation (eqn (17))17
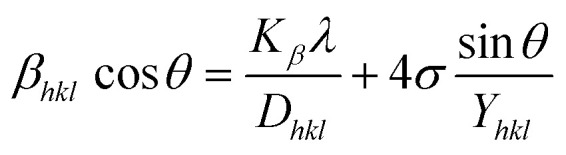


**Fig. 6:**
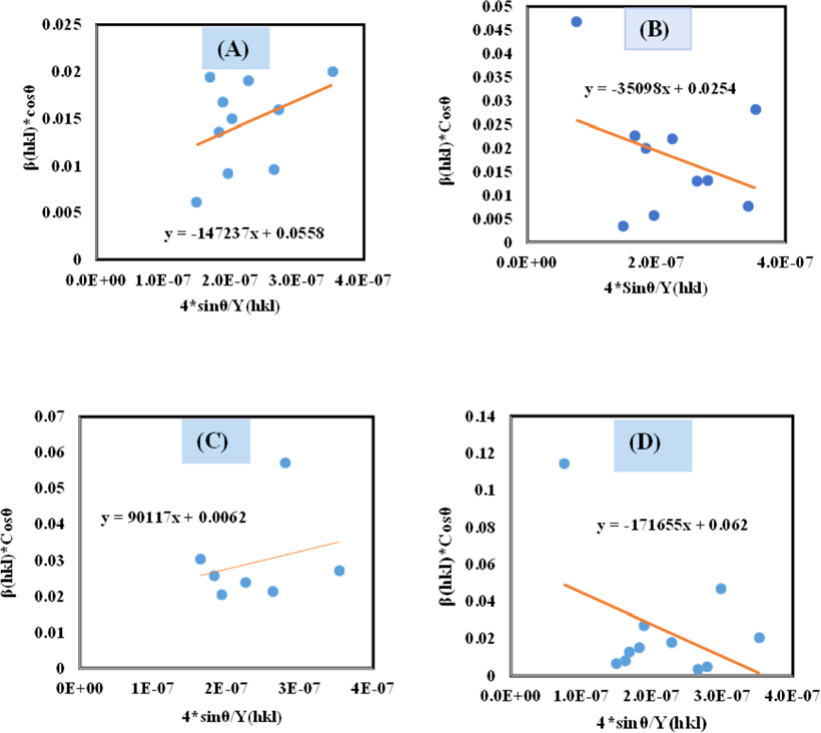
Determination of crystallite size by utilizing uniform stress deformation model for (A) Ca(OH)_2__HAp, (B) TiO_2__HAp, (C) CaCO_3__HAp and (D) ZnO_HAp.

Thus, plotting *β*_total_ × cos *θ* on the *Y*-axis as well as 4 × sin *θ*/*Y*_(*hkl*)_ along the *X*-axis produces a straight-line graph. The gradient of this expressed straight line delivers a measure of stress (*σ*), whilst the point of intersection offers the crystallite size *D*_(*hkl*)_ of the HAp nanocrystals. The plots are illustrated in Fig. 6, and the computed *σ* and *D*_(*hkl*)_ values are shown in Table 2.

#### 3.1.6 Uniform deformation energy density model (UDEDM)

UDM includes anisotropic entities that necessitate an alteration of the W–H relationship for effective alignment in anisotropic nanocrystals.^39^ UDEDM, determined by Hooke's law, demonstrates a linear relationship between *σ* and *ε* in real crystals. Yet, this straight proportionality is unsuitable since there are defects in the long-range order, including agglomerations and dislocations. UDEDM evaluates crystal imperfections, asymmetric deformation, and distortion causes as a measure of energy density (*u*), so the stress and strain constants remain independent.^40^Eqn (18) denotes *ε* (energy per unit volume), which is determined by Hooke's expression.18
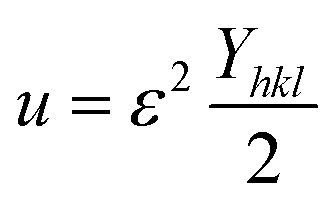


Reorganizing eqn (18) concerning *ε* and replacing it with eqn (14), the UDEDM equation is obtained as follows (eqn (19)).19
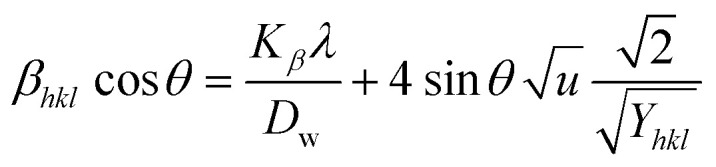


**Fig. 7:**
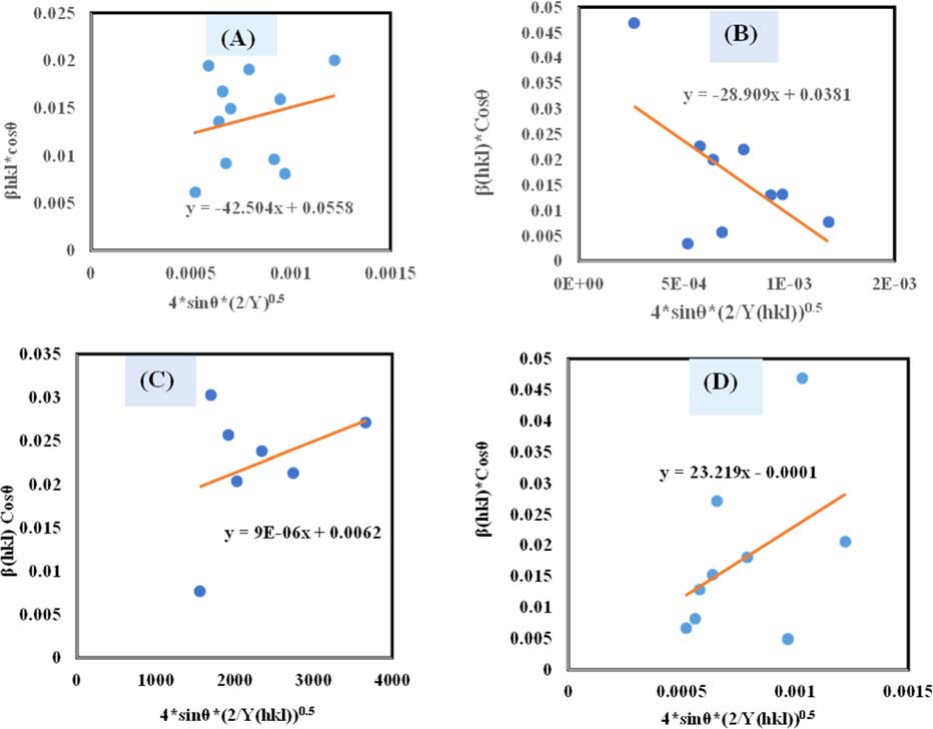
Determination of crystallite size by utilizing uniform deformation energy density model for (A) Ca(OH)_2__HAp, (B) TiO_2__HAp, (C) CaCO_3__HAp and (D) ZnO_HAp.

By plotting the graph between *β*_total_ cos *θ* on the *Y*-axis as well as 
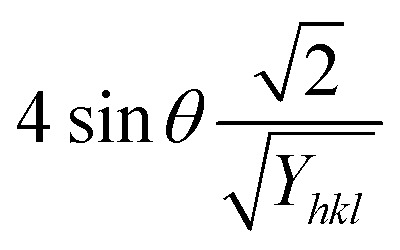
. On the *X*-axis, an anisotropic energy density (*u*) and crystallite size (*D*_w_) were measured from the slope and *Y*-intercept (Fig. 7). The estimated crystallite dimensions are shown in Table 2.

#### 3.1.7 Halder–Wagner method (HWM)

The SSP technique utilizes the Gaussian function to express the expansion of tension and the Lorentzian function to symbolize the broadening of size in XRD patterns. However, the XRD area correlates to the Gaussian operation, whereas the bottom drops excessively. The lower portion of the profile fits the Lorentz function yet does not match the XRD peak area.^41,42^ The Halder–Wagner approach employs the symmetrical Voigt function, a convolution of Gaussian and Lorentzian processes, to describe the FWHM of the physical profile, as illustrated in eqn (20).^42,43^

20 *β*_*hkl*_^2^ = *β*_L_*β*_*hkl*_ + *β*_G_^2^

where, *β*_L_ = FWHM for Lorentzian function. *β*_G_ = FWHM for Gaussian function. The approach lends a larger weight to Bragg peaks in small and intermediate angles, decreases reflection overlapping, and connects crystallite size and lattice *ε* with the H–W technique, as indicated by eqn (21)–(23).^34^21
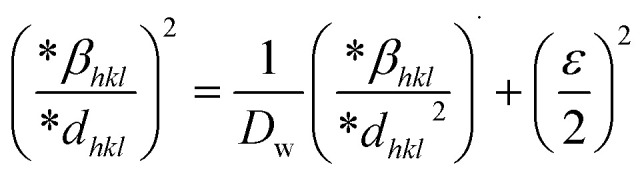
22
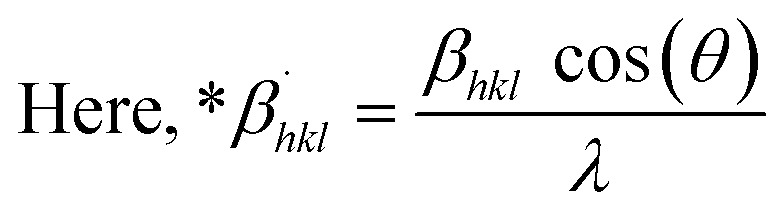
23
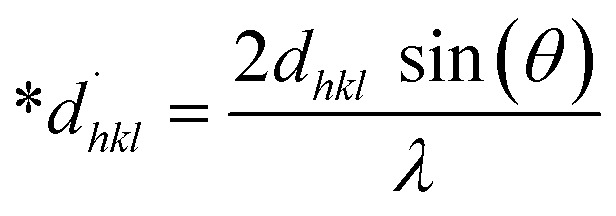


**Fig. 8:**
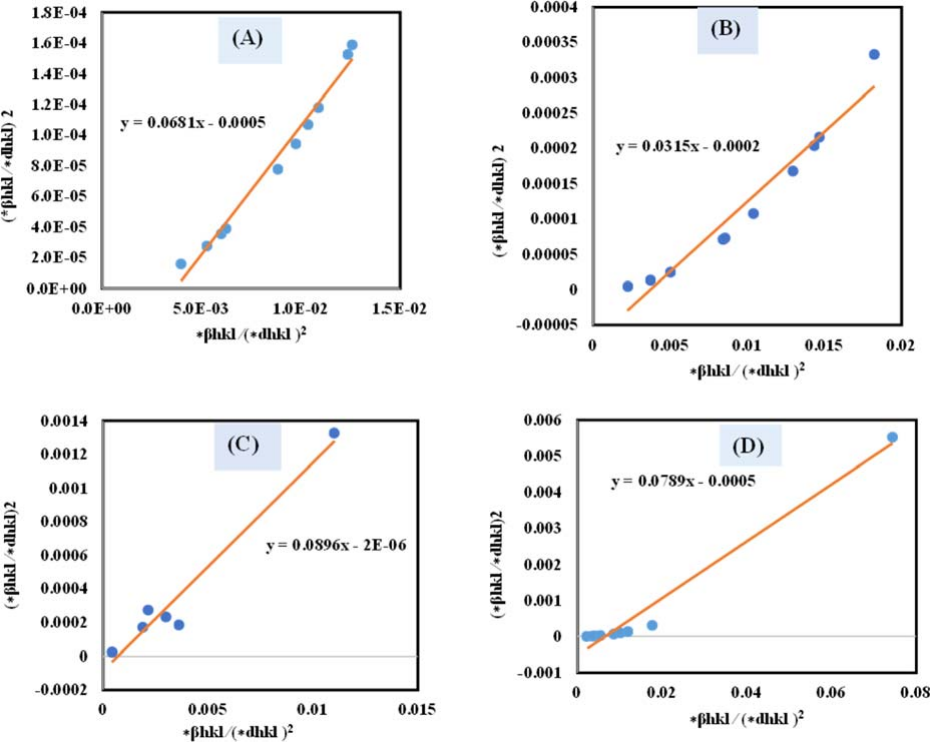
Determination of crystallite size by utilizing the Halder–Wagner model for (A) Ca(OH)_2__HAp, (B) TiO_2__HAp, (C) CaCO_3__HAp and (D) ZnO_HAp.

A plot of (*β_*hkl*_/*d_*hkl*_)^2^ on the *Y*-axis and *β_*hkl*_/(*d_*hkl*_)^2^ on the *X*-axis generates a straight line, where slope equal to 1/*D*_w_ and from the *y*-intercepts, microstrain was estimated (Fig. 8). The estimated crystallite size synthesized HAp is shown in Table 2.

### 3.2 Function group analysis

**Fig. 9:**
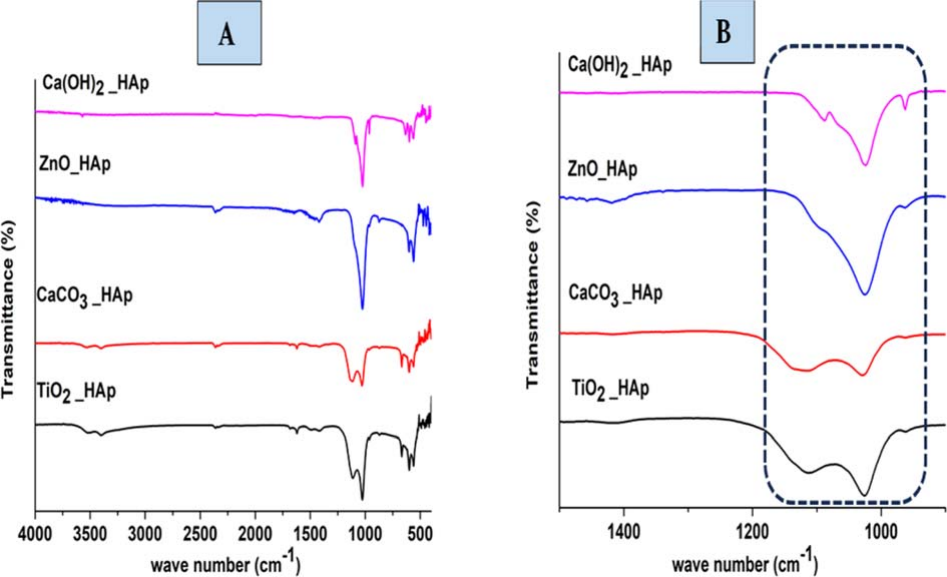
FTIR of synthesized pure and doped HAp (A) weave number 4000–400 cm^−1^ and (B) IR region of 1500–500 cm^−1^.

The functional group in the synthesized product is analyzed by Fourier Transform Infrared (FTIR) spectra (Model: IR-Prestige 21 (Shimadzu, Japan)), which are shown in Fig. 9. In HAp, PO_4_^3−^ and OH^−^ are optically active groups, which are responsible for the resulting spectra.^44^ In this present study, pure and doped HAp were synthesized through the wet chemical precipitation method to modify the crystalline structure, which also shows similar spectra. PO_4_^3−^ (Tetrahedral) ions possess four preliminary forms of vibrations, which are symmetric stretching (*ν*_1_), asymmetric stretching (*ν*_3_), symmetric bending (*ν*_2_), and asymmetric bending (*ν*_4_).^45^ Three forms of stretching oscillation were found around 962, 1026, and 1087 cm^−1^ wavenumbers, whereas bending vibration yielded peaks near 465, 563, and 599 cm^−1^ wavenumbers, which are equivalent to hydroxyapatite and the results have already been published.^46,47^ The peak at 962 cm^−1^ was observed due to (*ν*_1_) oscillation. Conversely, the peaks at 1087 and 1026 cm^−1^ were responsible for (*ν*_3_) vibration. At 563 and 599 cm^−1^ wavenumbers, (*ν*_4_) vibrations are observed, while at 473 cm^−1^ (*ν*_2_) vibration is predominant. Asymmetric bending (*ν*_4_) vibration is shown at 563 and 599 cm^−1^ wavenumbers, while symmetric bending (*ν*_2_) is observed at 473 cm^−1^ wavenumbers. In this experiment for synthesized HAp, OH^−^ shows FTIR peaks at 3000–3800 cm^−1^ wavenumber; these identical positions were reported in numerous literature.^48,49^

### 3.3 Optical properties

The spectral band gap of pure and doped HAp was evaluated utilizing a double-beam UV-vis spectrophotometer (Model: U-2910), wherein the powder sample was dispersed in water at room temperature. The absorption frequency of synthesized HAp is used to measure the optical band gap in the UV-vis spectrophotometer. For direct band gap analysis, the Tauc plot method was used, which is expressed in the mathematical eqn (24).^50,51^

**Fig. 10:**
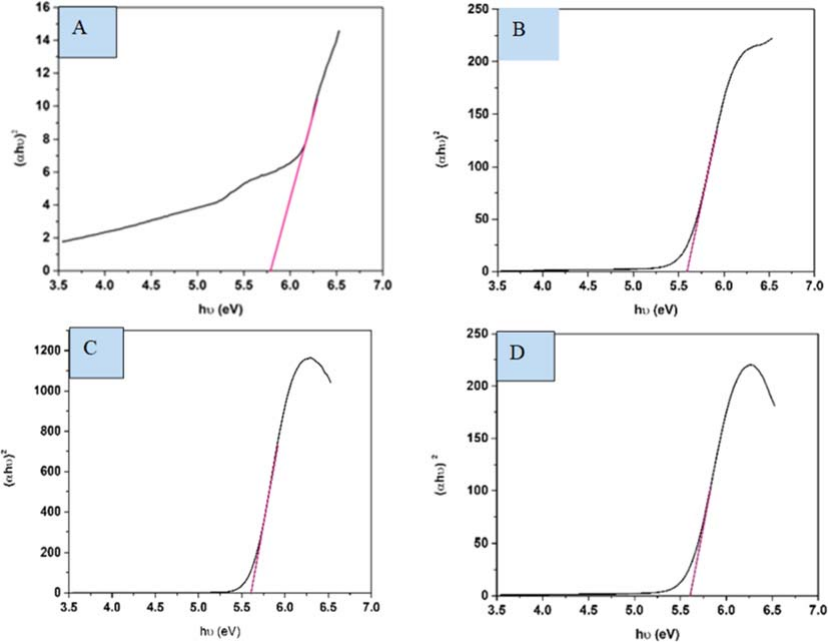
Optical band gap of (A) Ca(OH)_2__HAp, (B) TiO_2__HAp, (C) CaCO_3__HAp and (D) ZnO_HAp.

24 *αhμ* = *A*(*hθ* − *E*_g_)^*n*^

where, *h* is Planck's constant, *E*_g_ is the optical band gap. *A* is a constant, *α* is the absorption coefficient, *μ* is the photon frequency, as well as *n* = 1/2 for a direct band gap. The optical band gaps of the synthesized samples were estimated (Fig. 10) and grouped in Table 3.

**Table 3:** Estimated band gap of pure and synthesized HAp

Sample name	Optical band gap
Ca(OH)_2__HAp	5.77 eV
TiO_2__HAp	5.59 eV
CaCO_3__HAp	5.61 eV
ZnO_HAp	5.58 eV

### 3.4 Scanning electron microscopy (SEM)

**Fig. 11:**
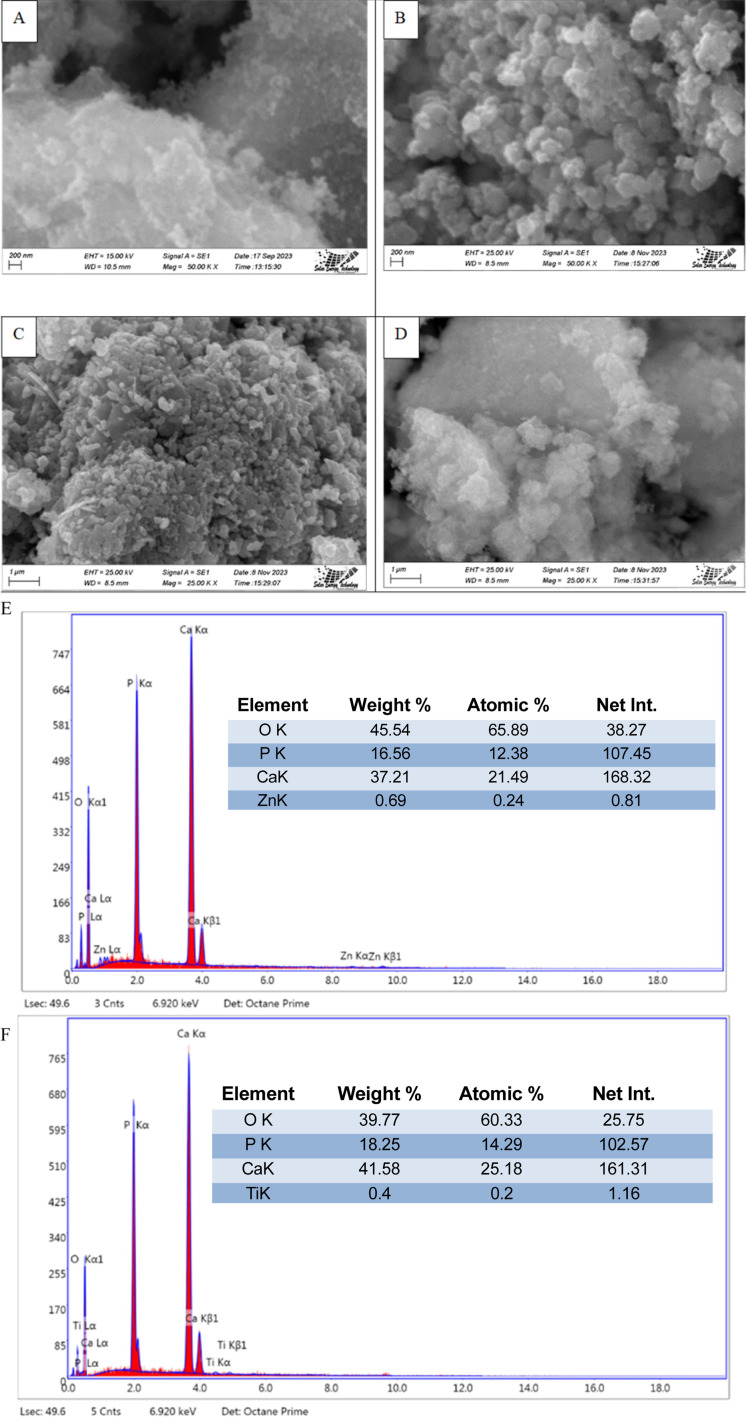
Scanning electron micrograph of synthesized HAp (A) TiO_2__HAp, (B) CaCO_3__HAp, (C) ZnO_HAp and (D) Ca(OH)_2__HAp. EDS with elemental mapping of synthesized HAp (E) TiO_2__HAp, and (F) ZnO_HAp.

The SEM images (Machine model: JEOL JSM-7610F) of synthesized HAp are shown in Fig. 11, where different types of dopants, as well as different precursors, are used for nanocrystalline HAp synthesis. From analyzing the images, it's prominently visible that many distinctive forms of nanoparticles are present in synthesized HAp, which tend to agglomerate. Apart from that, most of these particles are greater than a hundred nanometers as a prominent form. The presence of doped metal oxide was confirmed by the EDS analysis which is visualized in Fig. 11. The existences of Ti and Zn were found in the doped hydroxyapatite.

### 3.5 Pure and synthesize HAp photocatalytic activity

#### 3.5.1 Effect of contact time on CR degradation

**Fig. 12:**
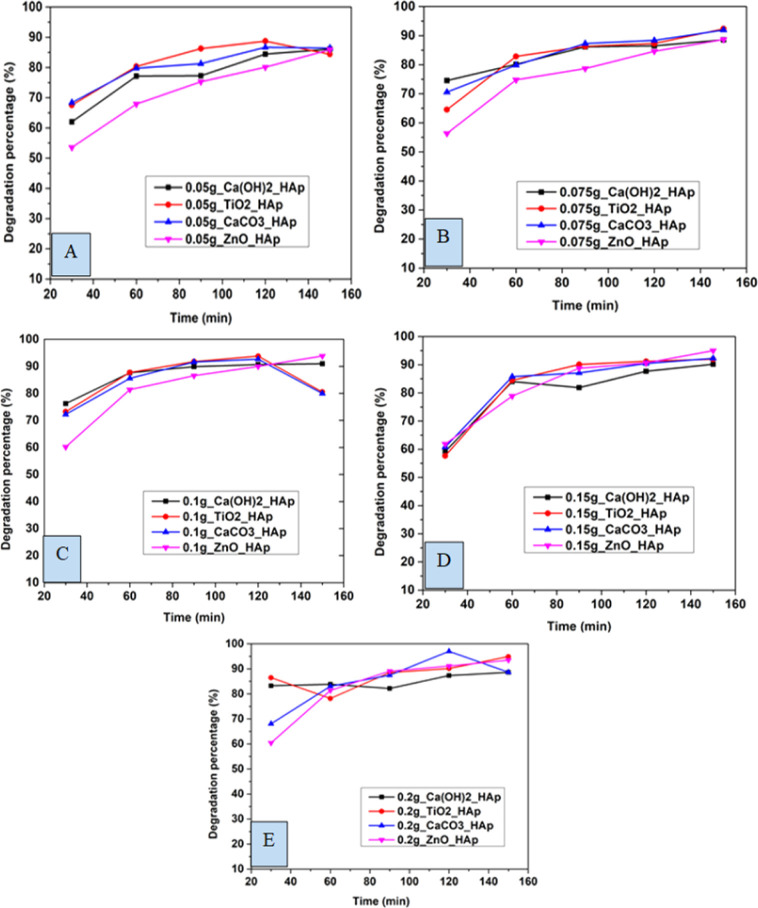
Degradation percentage of synthesized HAp in terms of several time laps for adsorbent (A) 0.05 g, (B) 0.075 g, (C) 0.1 g, (D) 0.15 g and (E) 0.2 g.

**Fig. 13:**
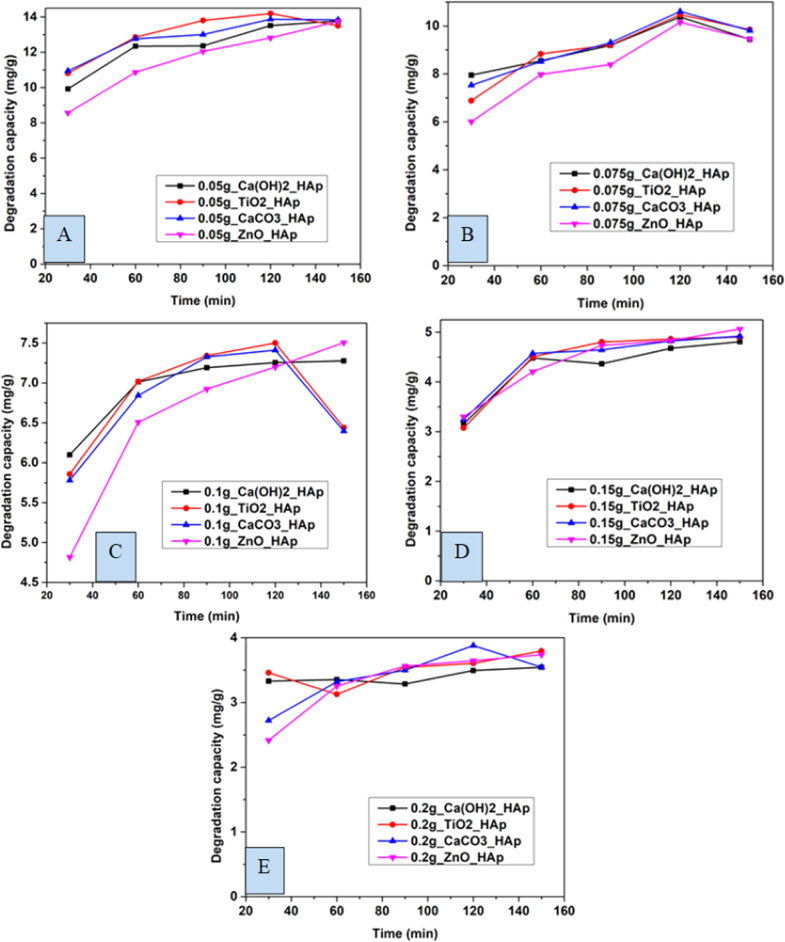
Degradation capacity of synthesized HAp in terms of several time laps for adsorbent (A) 0.05 g, (B) 0.075 g, (C) 0.1 g, (D) 0.15 g and (E) 0.2 g.

In this experiment, distinctive time frames such as (30, 60, 90, 120, and 150 min) and different amounts of adsorbent like 0.05 g, 0.075 g, 0.1 g, 0.15 g, 0.2 g were observed to estimate the degradation percentage as well as the capacity of CR solution (Fig. 12 and 13). The degradation percentage rises with the increase in adsorbent, as the number of active sites increases, hence boosting the effectiveness of the photocatalyst. The highest degradation percentage was observed when using 0.2 g of adsorbent and a contact time of 150 minutes. Conversely, the lowest degradation percentage is seen for 0.05 g of adsorbent at time 30 minutes. The optimum values for degradation percentage are considered at 0.1 g of adsorbent at 90 min, where 89%, 91%, 91%, and 86% degradation of CR are estimated for Ca(OH)_2__HAp, TiO_2__HAp, CaCO_3__HAp, and ZnO_HAp, correspondingly.

Similarly, the degradation capacity of the synthesized HAp is investigated for adsorbent 0.05 g, 0.075 g, 0.1 g, 0.15 g, and 0.2 g correspondingly. The optimum degradation capacity is considered for adsorbent 0.1 g and the contact time 90 minutes which are 7.19 mg g^−1^, 7.34 mg g^−1^, 7.32 mg g^−1^, 6.92 mg g^−1^for Ca(OH)_2__HAp, TiO_2__HAp, CaCO_3__HAp, and ZnO_HAp, respectively.

#### 3.5.2 Effect of catalyst dose on the photodegradation

**Fig. 14:**
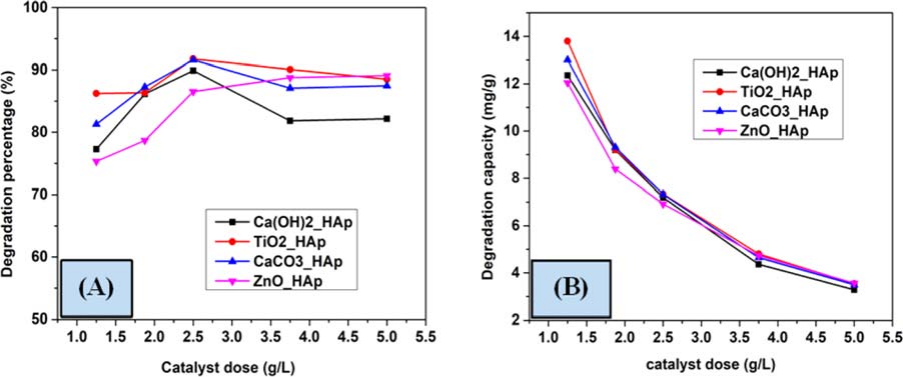
Effect of various doses on the photodegradation (A) degradation percentage and (B) degradation capacity.

To investigate the impact of catalytic dose on the photodegradation of CR, several compounds such as Ca(OH)_2__HAp, TiO_2__HAp, CaCO_3__HAp, and ZnO_HAp catalyst from 1.25 to 5 g L^−1^ at an initial concentration of CR as 2.87 × 10^−5^ M as well as irradiation time 90 minutes were employed. Fig. 14 denotes the alteration in the photodegradation of CR as the catalyst at several dosages of the above-mentioned samples. In the case of Ca(OH)_2__HAp, TiO_2__HAp, and CaCO_3__HAp, the observed % of removal of CR is vindicated to rise within catalyst dosage up to 2.5 g L^−1^ and extended their height values as 89.89%, 91.76% and 91.58% correspondingly. This phenomenon can be ascribed to an increased formation in accessible surface area for the photocatalysts, facilitating the generation of more active radicals.^28,52,53^ Conversely, with the additional enhancement in the dosage of the catalyst from 2.5 to 5 g L^−1^, the percentage of degradation efficacy was attenuated. However, this could be attributed to the high amount of catalytic dose, which makes the solution turbid and reduces the photodegradation of the solution.^54,55^ Additionally, for ZnO_HAp, the degradation percentage rises with the increase in catalyst dosage, which is around 90% for 5 g L^−1^.

#### 3.5.3 Effect of solution pH the photodegradation

**Fig. 15:**
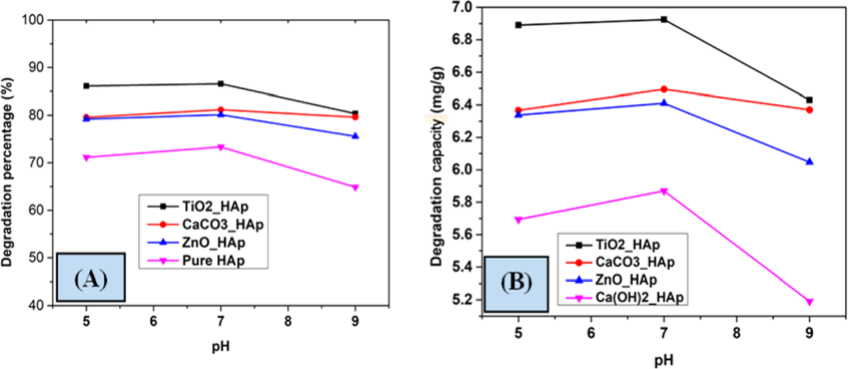
Impact of pH on photodegradation (A) degradation percentage and (B) degradation capacity.

A group of investigations was accomplished to investigate the effect of pH on the degradation percentage and degradation capacity of CR dye. Different pH, such as pH 5, pH 7, and pH 9 were studied to determine the effect of varying pH (Fig. 15). A 40 mL solution of 20 mg L^−1^ concentration of CR dye was irradiated at ambient temperature under a 500 W halogen lamp for 90 minutes. The pH of the reaction solution substantially influences photocatalytic degradation, as it effectively streamlines the entire process. However, estimating the effect of solution pH on photodegradation is a crucial event; several variables have been identified affecting this, such as the electrostatic and catalyst's nature and the presence of the pollutant molecule.^56,57^ Lower pH levels are observed to increase the photodegradation of weakly acidic contaminants, contradicting previous study results.^58,59^ The value of degradation percentage increased with increasing pH from 5 to 7 but decreased at pH 9. The reaction was greatly impacted by the numerous hydroxyl and hydrogen ions, resulting in a larger expenditure owing to the requirement for more effort and an acidic solution; hence, pH 7 was picked as the optimal value.

#### 3.5.4 Effect of initial CR concentration on photodegradation

**Fig. 16:**
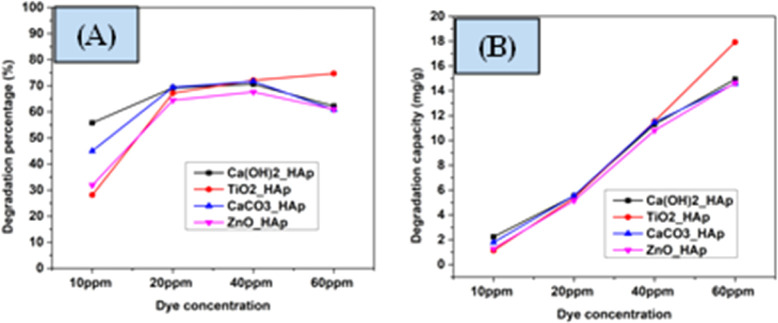
Effect of CR concentration on photodegradation (A) degradation percentage (B) degradation capacity.

The concentration of pollutants possesses a crucial role in photocatalytic degradation, and investigations were done to establish the appropriate dye concentration for optimal effects. For pH 7 and 2.5 g L^−1^ of catalyst dose, the impact of various dye concentrations was analyzed. In Fig. 16, the percentage of degradation as well as degradation capacity are denoted. From the analysis, it's unequivocally stated that the degradation percentage increases within the dye concentration raised from 10 mg L^−1^ to 40 mg L^−1^, which can be correlated to the manner that the extent of a suitable number of active sites as well as the radicals are present on the synthesized HAps surface. Furthermore, the additional expansion in initial dye concentration above 40 mg L^−1^ results in a decrease in the efficacy of degradation percentage. The removal of CR dye diminishes with increasing dye concentration owing to the capture of more photons by the dye compared to the photocatalyst, which produces a low amount of hydroxyl radicals.^60^ The number of active sites produces more active radicals O_2_˙^−^ as well as ˙OH, which maximize the efficiency of photocatalytic event. Conversely, the reduction in the degradation percentage are observed when the number of free radicals becomes low. In comparison to the degradation percentage, the degradation capacity increased by increasing the concentration of the dye solution.

#### 3.5.5 Photocatalytic mechanism of HAp

**Fig. 17:**
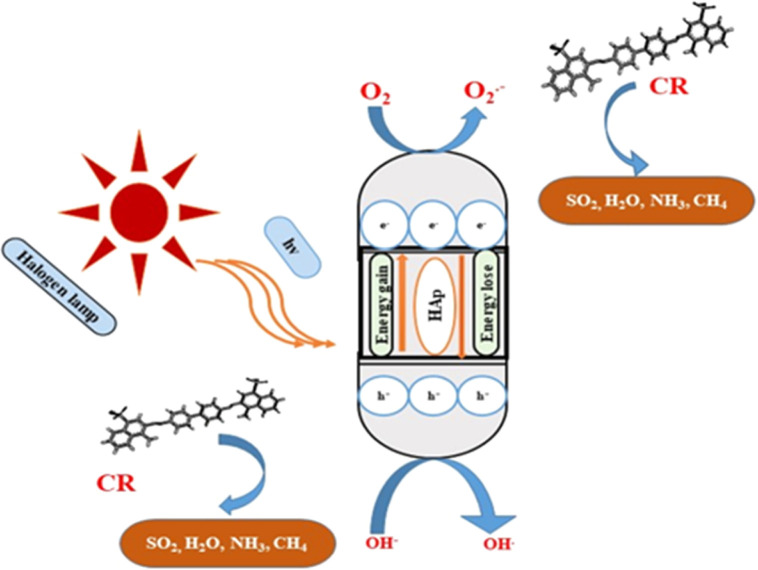
Possible photocatalytic pathway of hydroxyapatite.

The photodegradation technique employs a photon source to activate a redox reaction, enhancing the efficacy of the process by limiting recombining properties.^51^ The proposed simplified reaction mechanism for Congo red degradation using pure, TiO_2_ as well as ZnO doped HAp is illustrated in Fig. 17 and mathematically expressed in eqn (25)–(32).25
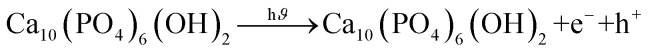
26
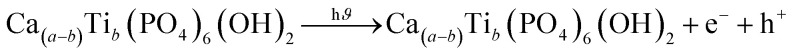
27
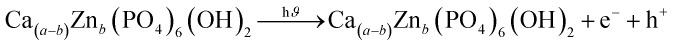

28 Ca_10_(PO_4_)_6_(OH_2_)/Ca_(*a*−*b*)_Ti_*b*_(PO_4_)_6_(OH)_2_/Ca_(*a*−*b*)_Zn_*b*_(PO_4_)_6_(OH)_2_ + e^−^ + h^+^ → ˙O_2_ + ˙OH˙ + ˙OH^−^

29 H_2_O + h^+^ + e^−^ → H^+^ + ˙O_2_ + OH^−^ + ˙OH^−^ + ˙O_2_^−^

30 ˙OH^−^ + ˙OH^−^ → H_2_O_2_

31 h^+^ + H_2_O_2_ + e^−^ → ˙OH* + ˙OH^−^

32 ˙OH^−^ + ˙OH* + ˙O_2_^−^ + ˙O_2_ + e^−^ + h^+^ + Congo red dye → Intermediate → H_2_O + CO_2_

Doping hydroxyapatite (HAp) with materials like TiO_2_ and ZnO alters its electronic and optical properties, particularly its band gap, enhancing photocatalytic activity. This process creates defects and vacancies in the HAp lattice, facilitating electron–hole pair separation and improving reaction efficiency. The effect of doping on HAp's band gap and photocatalytic activity is complex and depends on factors like dopant type, concentration, synthesis method, and application. Direct band gap materials are preferred due to their prolonged lifetime of charge carriers, while indirect band gap materials may have advantages, but their direct band gap is inherent to HAp.^61–64^

Photocatalytic agents, such as free radicals and electrons, play a crucial part in the photocatalytic deterioration of Congo red dye. These agents, coupled with holes, act as effective oxidizing agents, helping in the formation of more reactive species owing to the delayed reproduction of e^−^ as well as h^+^. This phenomenon can be mathematically expressed with the help of Mulliken's theory eqn (33) and (34).^65^

33 *E*_CB_ = *X* − *E*_c_ − 0.5*E*_bg_

34 *E*_VB_ = *E*_CB_ + *E*_bg_

In these equations, *E*_CB_ (energy of conduction band), *X* (electronegativity of the photocatalyst), *E*_c_ (free electron energy and its magnitude is 4.5 eV), *E*_bg_ (band gap energy), and *E*_VB_ (valence band energy), correspondingly. Synthesized HAp possesses electronegativity as 5.89 eV, which is attributable to the geometrical mean of its structure, depicted in literature.^65^ The estimated magnitude of the conduction band, as well as the valence band, are shown in Table 4. The Synthesized HAp demonstrated greater negativity potentials of the Conduction band than O_2_/˙O_2_^−^ (−0.33 eV) as well as greater positive potentials of the valence band compared to OH/˙OH (1.99 eV), showing both radicals can be generated for photocatalysis of the Congo red dye.

**Table 4:** Synthesized HAp Valance Band (VB) as well as Conduction Band (CB) potentials for

Sample name	Conduction band	Valance band
Ca(OH)_2__HAp	−1.495	4.275
TiO_2__HAp	−1.4	4.18
CaCO_3__HAp	−1.415	4.195
ZnO_HAp	−1.4	4.18

#### 3.5.6 Photocatalytic reusability experiments

**Fig. 18:**
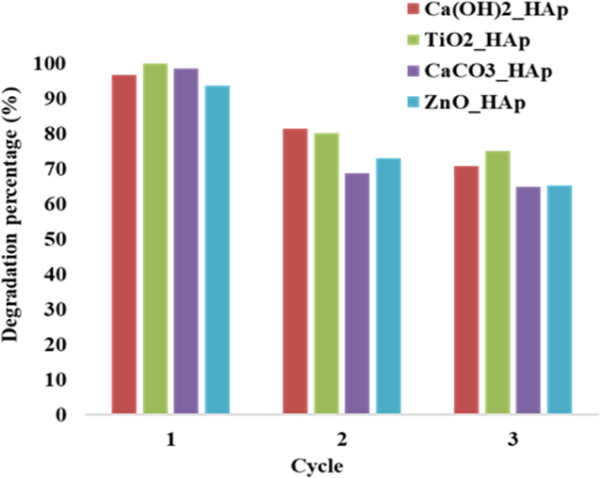
Reusability of synthesized HAp in terms of degradation percentage.

The recyclability or reusability test is a vital criterion that measures a catalyst's optimum and repeated usage, assuring its long-term sustainability.^28^Fig. 18. Denotes the recyclability test of synthesized pure and dope HAp at optimum parameters like 0.1 g of catalyst, 2.87 × 10^−5^ M, 40 mL CR solution, at 90 minutes under halogen lamp (500 W) for three cycle tests.

The dye solution was filtered and dried at 60 °C in an oven for 2 hours, and then the new solution of CR dye was introduced to the dried samples. From Fig. 18, it's prominently visible that the degradation percentage for the first cycle, Ca(OH)_2__HAp, and ZnO_HAp, shows a lower degradation percentage as compared to TiO_2__HAp as well as CaCO_3__HAp. In the second cycle, ZnO_HAp exhibited a cabalistic decrease in degradation, while CaCO_3__HAp depicted the lowest degradation percentage. Finally, in the third cycle, CaCO_3__HAp and ZnO_HAp had lower percentages of degradation as compared to the Ca(OH)_2__HAp and TiO_2__HAp, whereas Ca(OH)_2__HAp showed lower degradation. This phenomenon of adsorption of dye molecules on the photocatalyst surface can be described as the extent of unoccupied active sites present in the photocatalyst. A greater amount of photodegradation efficiency is observed where the dye molecules are easily adsorbed by active sites staying in the samples. Since the quantity of active sites gets more engrossed within the cycles, this causes lower photocatalytic degradation in the subsequent cycle.

#### 3.5.7 Scavenging studies

**Fig. 19:**
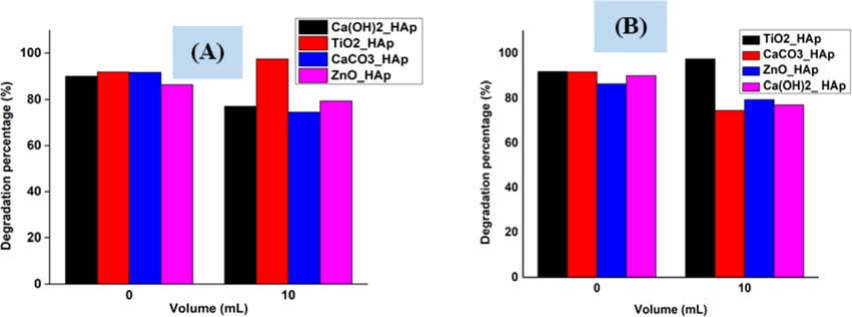
Scavengers effect on the degradation of Congo red dye (A) IPA (10 mL) and (B) EDTA (10 mL).

2-Propanol (IPA) and EDTA were used as the scavenging agents for OH* and electrons correspondingly. Fig. 19 shows a scavenging test that depicts 2-propanol and EDTA, considerably decreased degradation rate, indicating the OH* and electrons are preliminary photoactive species related to photodegradation of CR dye. Conversely, in both scavenging tests, TiO_2__HAp shows a different phenomenon where degradation percentage increased while adding IPA as well as EDTA, which can be the reason for having holes as a predominant.

## 4 Kinetics study

**Fig. 20:**
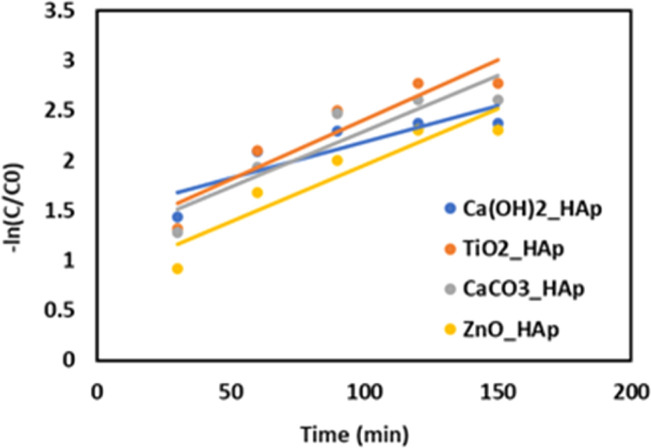
The plot of −ln(*C*/*C*_0_) against time (min) for various samples to estimate the reaction rate constant for 0.1 g of photocatalyst.

The photodegradation kinetics (Fig. 20) of CR by Ca(OH)_2__Hap, TiO_2__Hap, CaCO_3__Hap, and ZnO_Hap have been studied under simulated sunlight. Previous research indicates that the rate of photocatalytic degradation of organic molecules is affected by the initial solution concentration and is described by pseudo-first-order kinetics, which is further elaborated in the Langmuir–Hinshelwood model to account for solid–liquid interface interactions.^66–69^ The degradation rate of dyes may be stated as:

35 ln(*C*/*C*_0_) = −*K*_1_*t*

where, *C*_0_ = initial concentration of reactant (mol L^−1^), and *C* = final concentration of reactant (mol L^−1^). The first-order rate constant (*K*_1_) was estimated by plotting time (*t*) along the *x*-axis and −ln(*C*/*C*_0_) along the *y*-axis, and generated graph is shown in Fig. 20. Table 5 provides a detailed record of the rate constant (*k*_1_) and regression coefficient (*R*^2^).

**Table 5:** Estimated values of linear fit for synthesized HAp of 0.1 g photo catalyst dose

Sample name	Intercept	Intercept	Slope	Slope	Statistics
	Value	Standard error	Value	Standard error	Adj. *R*-square
Ca(OH)_2__HAp	1.4666	0.2426	0.0071	0.0024	0.6576
TiO_2__HAp	1.2156	0.2748	0.0119	0.0027	0.8161
CaCO_3__HAp	1.1824	0.2795	0.0111	0.0028	0.7853
ZnO_HAp	0.8263	0.2559	0.0112	0.0025	0.8201

The research data reveals a correlation between the reaction rate and the catalyst composition. The first-order rate constant values range from 0.82636 min^−1^ (ZnO_HAp) to 1.46661 min^−1^ (Ca(OH)_2__HAp), with an average of 1.172755 min^−1^. The lower rate constant indicates a slower reaction compared to pure HAp, while a higher rate constant indicates a faster reaction. A correlation coefficient closer to 1 indicates a strong positive correlation, indicating a significant impact of catalyst composition on reaction rate.^62,70–72^

### 4.1 Practical applications of this research

The laboratory scale study analyzed hazardous compounds in industrial wastewater, highlighting the need for further data on treating real-world wastewater containing a blend of organic and inorganic compounds. This study can be replicated using various pollutant types, including caprolactam, phenol, benzoic acid, toluene, adipic acid, anionic and cationic dyes, benzene, amoxicillin, and ciprofloxacin, but requires extensive literature study and a pilot plant study before industrial application.

## 5 Conclusion

Hydroxyapatite (HAp) was synthesized successfully in pure and metal-oxide-doped form and crystallite size, calculated from various models, carried good evidence for the formation of nano-sized products. The effectiveness of these synthesized materials as potential photocatalysts was evaluated, with factors such as contact time, catalyst dose, initial dye concentration, pH, radical scavengers, and catalyst reusability influencing activity, and found these materials can be applied as potential photo-catalysts for degradation of organic pollutants. The synthesized products maintained excellent catalytic activity even after three reuse cycles. TiO_2_-doped HAp showed 90% degradation of Congo red dye, suggesting it could be a potential candidate for synthetic dye degradation compared to pure and ZnO-doped HAp. It may also be applied for the degradation of growing contaminants in pharmaceutical wastewater, which is composed of most antibiotics. Overall, TiO_2_-doped HAp has significant potential in photocatalysis applications, and further research can be performed for industrial scale application.

## Data availability

Data will be made available on request.

## Author contributions

Md. Kawsar synthesized and characterized the hydroxyapatites, executed the photocatalytic experiment, and wrote the draft and original manuscript. Md. Sahadat Hossain conceived and designed the experiment and analyzed the data. Sumaya Tabassum executed the photocatalytic experiment along with Md. Kawsar. Newaz Mohammed Bahadur and Samina Ahmed supervised the findings of this work. Samina Ahmed supervised the overall work and managed the required facilities.

## Conflicts of interest

There are no conflicts to declare.

## Supplementary Material
